# Supplementary material for: Sequential roles for red blood cell binding proteins enable phased commitment to invasion for malaria parasites
Source: Nat Commun. 2023 Aug 1;14:4619. doi: 10.1038/s41467-023-40357-z (PMC10393984; doi:10.1038/s41467-023-40357-z)
Supplement: Supplementary file 1 — Supplementary Information [file 41467_2023_40357_MOESM1_ESM.pdf]

Sequential roles for red blood cell binding proteins enable phased commitment to invasion for malaria parasites

Melissa N. Hart[1,2], Franziska Mohring[1], Sophia M. Donvito[1], James A. Thomas[1], Nicole Muller-Sienerth[3], Gavin J. Wright[3,4], Ellen Knuepfer[2,5] Helen R. Saibil[6], Robert W. Moon[1]

Affiliations

1. Department of Infection Biology, Faculty of Infectious and Tropical Disease, London School of Hygiene and Tropical Medicine, London, WC1E 7HT, UK.
2. Department of Pathobiology and Population Sciences, Royal Veterinary College, Hawkshead Lane, Hatfield, AL9 7TA, UK
3. Wellcome Sanger Institute, Hinxton, Cambridge CB10 1SA, UK.
4. Department of Biology, Hull York Medical School, York Biomedical Research Institute, University of York, Wentworth Way, York, YO10 5DD, UK.
5. Malaria Parasitology Laboratory, Francis Crick Institute, London, NW1 1AT, UK
6. ISMB, Biological Sciences, Birkbeck, University of London, Malet St, London WC1E 7HX

Supplementary Data

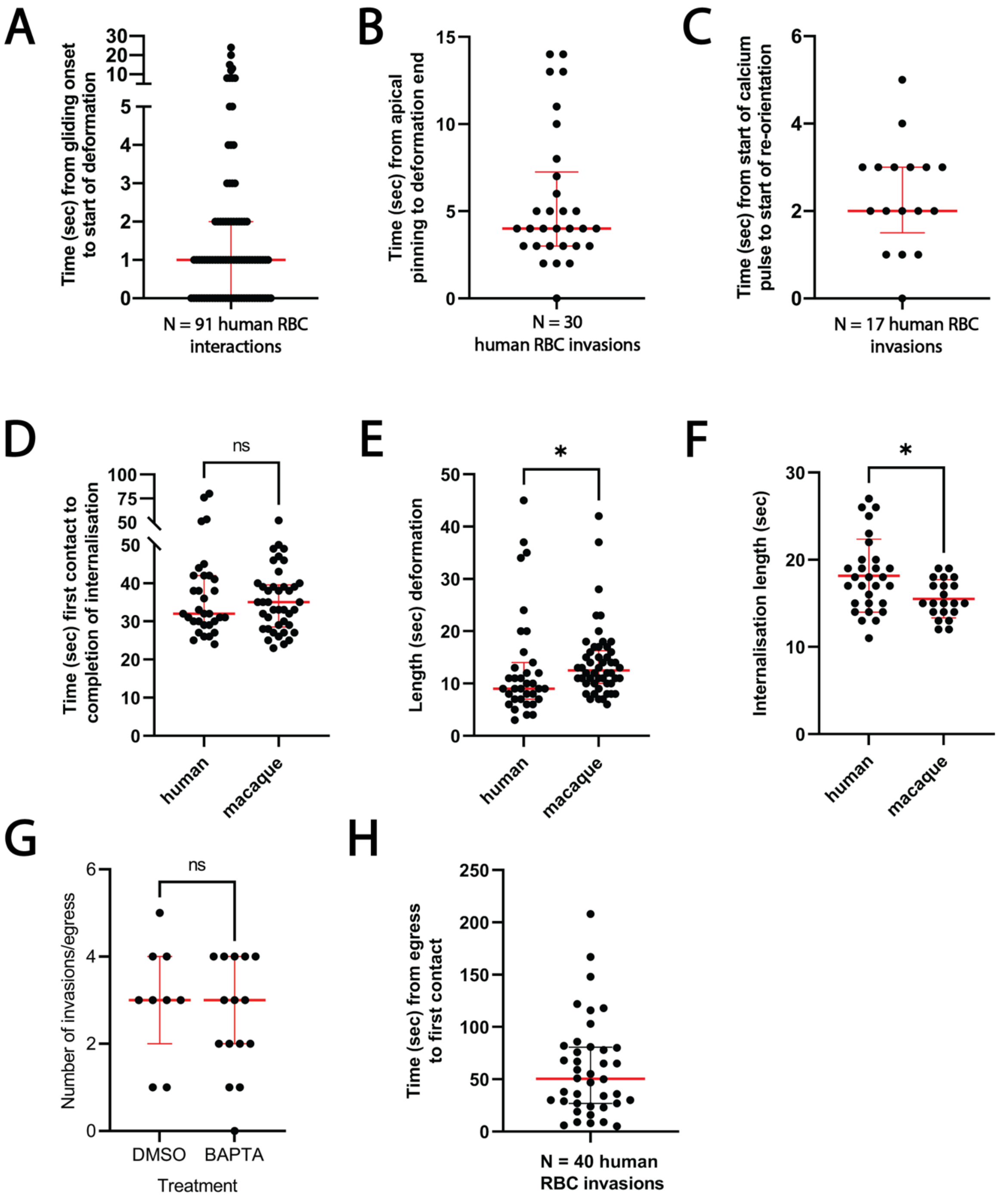

Supplementary Figure 1. Analysis of *P. knowlesi* interactions with human and macaque RBCs
(A) Merozoites spend a median of 1 second gliding on human RBCs (N = 91 interactions: IQR = 2 sec). **(B)** Merozoites cease forward movement across human RBC surfaces a median 4 sec (IQR = 4.25 sec) before deformation subsides. N = data from 30 invasions. **(C)** Merozoites re-orientate on human RBC surfaces a median 2 sec (IQR = 3 sec) after a calcium pulse is observed. N = data from 17 invasions. **(D)** There is no significant difference between the length of time from first contact to completion of internalisation for human (median = 32 sec; IQR = 12.75 sec; N = 32 invasions) vs. macaque invasions (median = 35 sec; IQR = 11 sec; N = 41 invasions). 'Ns' indicates p = 0.976, when analysed using a two-tailed Mann-Whitney U-test. **(E)** *P. knowlesi* merozoites spend significantly longer deforming macaque RBCs (median = 12.5 sec; IQR = 6.3 sec; N = 50 invasions) vs. human (median = 9 sec; IQR = 77 sec; N = 35 invasions) prior to internalisation. * indicates p = 0.024, when analysed using a two-tailed Mann-Whitney U-test. **(F)** *P. knowlesi* merozoites spend significantly longer actively invading human RBCs (mean = 18.2 sec; SD = 4.2 sec; N = 29 invasions)

vs. macaque (mean = 15.5 sec; SD = 2.2 sec; N = 21 invasions). * indicates p = 0.011, when analysed using two-tailed unpaired t-test. **(G)** Merozoites invade BAPTA-AM treated human RBCs (median = 3 invasions/egress; IQR = 2 invasions/egress; N = 15 egresses) with equal efficiency to control (median = 3 invasions/egress; IQR = 2 invasions/egress; N = 9 egresses). 'Ns' indicates p = 0.544, as determined using a two-tailed Mann-Whitney U-test. **(H)** Merozoites typically contact the human RBC they will invade within a median 50.5 sec post egress (IQR = 53.8 sec). For all graphs, error bars depict median and IQR.

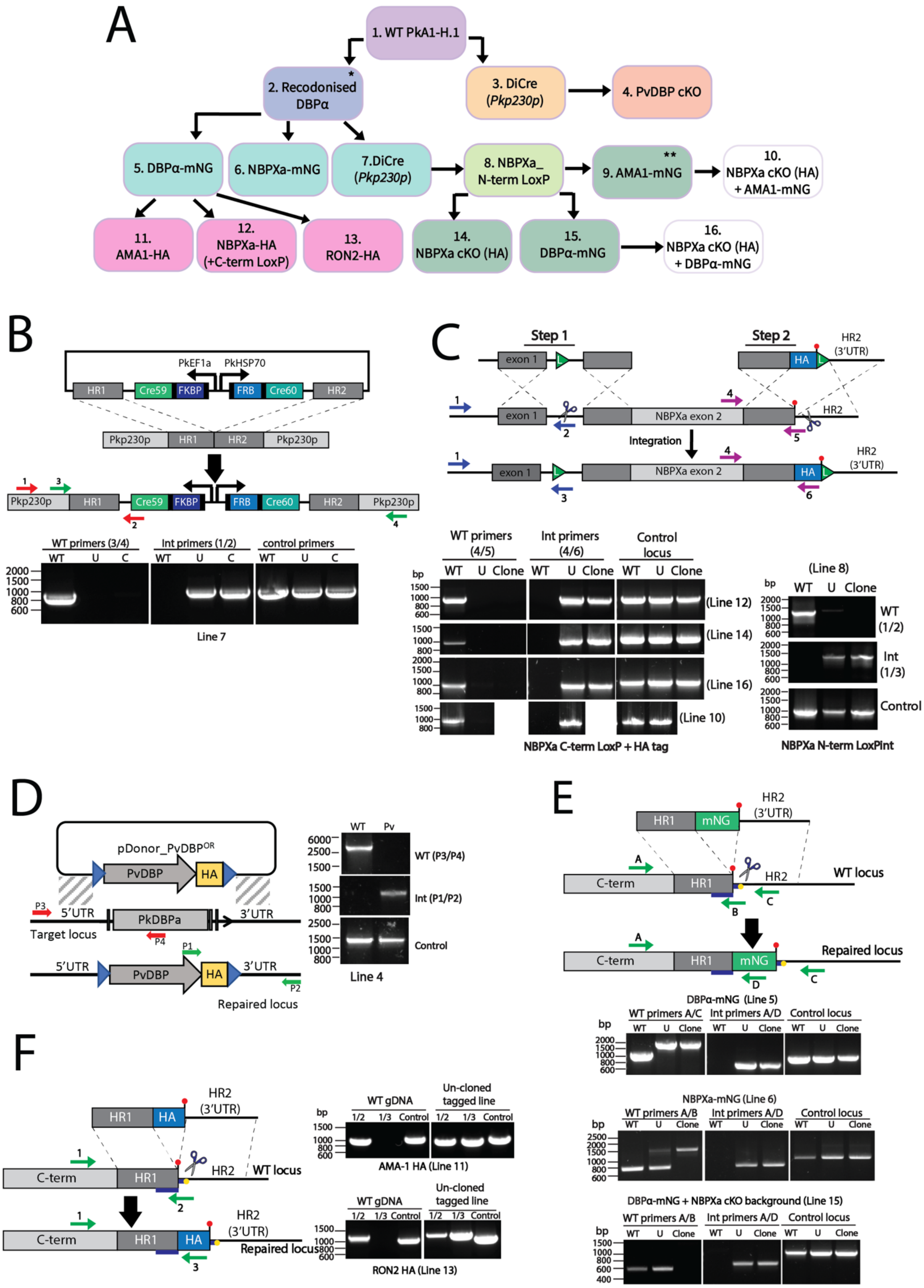
A
1. WT PkA1-H.1
2. Recodonised DBPα
3. DiCre (Pkp230p)
4. PvDBP cKO
5. DBPα-mNG
6. NBPXa-mNG
7.DiCre (Pkp230p)
8. NBPXa_ N-term LoxP
9. AMA1-mNG
10. NBPXa cKO (HA) + AMA1-mNG
11. AMA1-HA
12. NBPXa-HA (+C-term LoxP)
13. RON2-HA
14. NBPXa cKO (HA)
15. DBPα-mNG
16. NBPXa cKO (HA) + DBPα-mNG
B
PkEF1a
PkHSP70
HR1
Cre59
FKBP
FRB
Cre60
HR2
Pkp230p
WT primers (3/4)
Int primers (1/2)
control primers
WT U C
2000
1500
1000
800
600
Line 7
C
Step 1
Step 2
exon 1
NBPXa exon 2
HA
HR2 (3'UTR)
Integration
WT primers (4/5)
Int primers (4/6)
Control locus
WT U Clone
bp
(Line 12)
(Line 14)
(Line 16)
(Line 10)
NBPXa C-term LoxP + HA tag
(Line 8)
WT (1/2)
Int (1/3)
Control
NBPXa N-term LoxPint
D
pDonor_PvDBP^OR
PvDBP
HA
5'UTR
3'UTR
PkDBPa
Target locus
P1
P2
P3
P4
Repaired locus
WT
Pv
6000
2500
1500
1000
800
WT (P3/P4)
Int (P1/P2)
Control
Line 4
E
HR1
mNG
HR2 (3'UTR)
C-term
WT locus
Repaired locus
DBPα-mNG (Line 5)
WT primers A/C
Int primers A/D
Control locus
NBPXa-mNG (Line 6)
WT primers A/B
DBPα-mNG + NBPXa cKO background (Line 15)
F
HR1
HA
HR2 (3'UTR)
C-term
WT locus
Repaired locus
WT gDNA
Un-cloned tagged line
1/2
1/3
Control
AMA-1 HA (Line 11)
RON2 HA (Line 13)

Supplementary Figure 2. Generation and genotyping of *P. knowlesi* transgenic lines (A) Flow chart depicting transgenic lines generated/used in this study. *indicates line described in[1]. **indicates line described in[2]. **(B)** Generation of Dimerisable Cre Recombinase (DiCre) expressing background lines (lines 3 and 7). Primers FM49/FM50 (positions 3/4 in schematic) detect WT parasites and primers FM90/FM32 (positions 1/2) detect transgenic parasites. **(C)** Generation of NBPXa cKO/HA tagged lines. Lines were generated with two steps. Step 1 = integrating LoxP sequence into the endogenous NBPXa intron. Primers MH959/MH1143 (Positions 1/2 in schematic) detect WT parasites. Primers MH959/MH1172 (positions 1/3) detect transgenic parasites. Step 2 = integrating LoxP sequence + single HA tag at NBPXa C-term. Primers MH1129/MH1128 (positions 4/5) detect WT parasites, and primers MH1129/MH563 (positions 4/6) detect transgenic parasites. **(D)** Generating a PvDBP cKO line. WT PkDBPα was swapped with a floxed PvDBP sequence. Primers FM1973/FM188 (positions 3/4 in schematic) detect WT parasites. Primers FM1897/ FM1041 (positions 1/2) detect transgenic parasites. 'Pv' = transgenic clone. **(E)** Schematic showing insertion of mNeonGreen (mNG) tag prior to the stop codon (red circle) of *Pk* invasion genes. Blue lines indicate the position of the Cas9 guide sequences. For DBPα mNG, line 5, primers FM421/FM225 detect WT parasites (positions A/C in schematic). Primers FM421/MH291 detect transgenic parasites (positions A/D). For DBPα mNG, line 15, primers FM421/MH1130 detect WT parasites (positions A/B). Primers FM421/MH291 detect integrated parasites, as above. For NBPXa mNG (line 6), primers MH835/1128 detect WT parasites (positions A/B). Primers MH835/MH291 (positions A/D) detect transgenic parasites. For all PCRs, 'U' = uncloned parasites. **(F)** Schematic showing generation of HA-tagged PkAMA-1 and PkRON2. For AMA-1, primers MH892/MH904 detect WT parasites (positions 1/2 in schematic). Primers MH892/MH563 detect transgenic parasites (positions 1/3). For RON2, primers MH890/MH909 detect WT parasites (positions 1/2). Primers MH890/MH563 detect transgenic parasites (positions 1/3). All primer sequences can be found in supplementary table 2. Expected PCR band sizes can be found in supplementary Table 1.

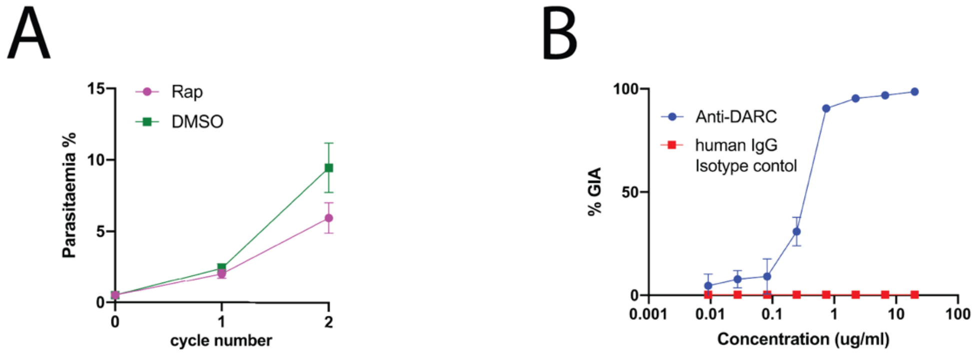

Supplementary Figure 3. Control growth assay data (A) Treating WT ring-stage *P. knowlesi* parasites with 10 nM rapamycin for 3hrs induces a slight, but non-significant growth defect (p = 0.122, when comparing final parasitaemias by a two tailed, unpaired t-test). Means of 5 independent assays shown. Error bars depict SEM. **(B)** Representative growth inhibition assay (GIA) results showing dose dependent inhibition of *P. knowlesi* invasion of human RBCs with increasing concentrations of human anti-DARC, Fy6 antibody (2C3 clone, Absolute antibody). EC50 = 0.3 μg/ml (range = 0.23-0.36 μg/ml, from 4 independent experiments). Mean and SD plotted.

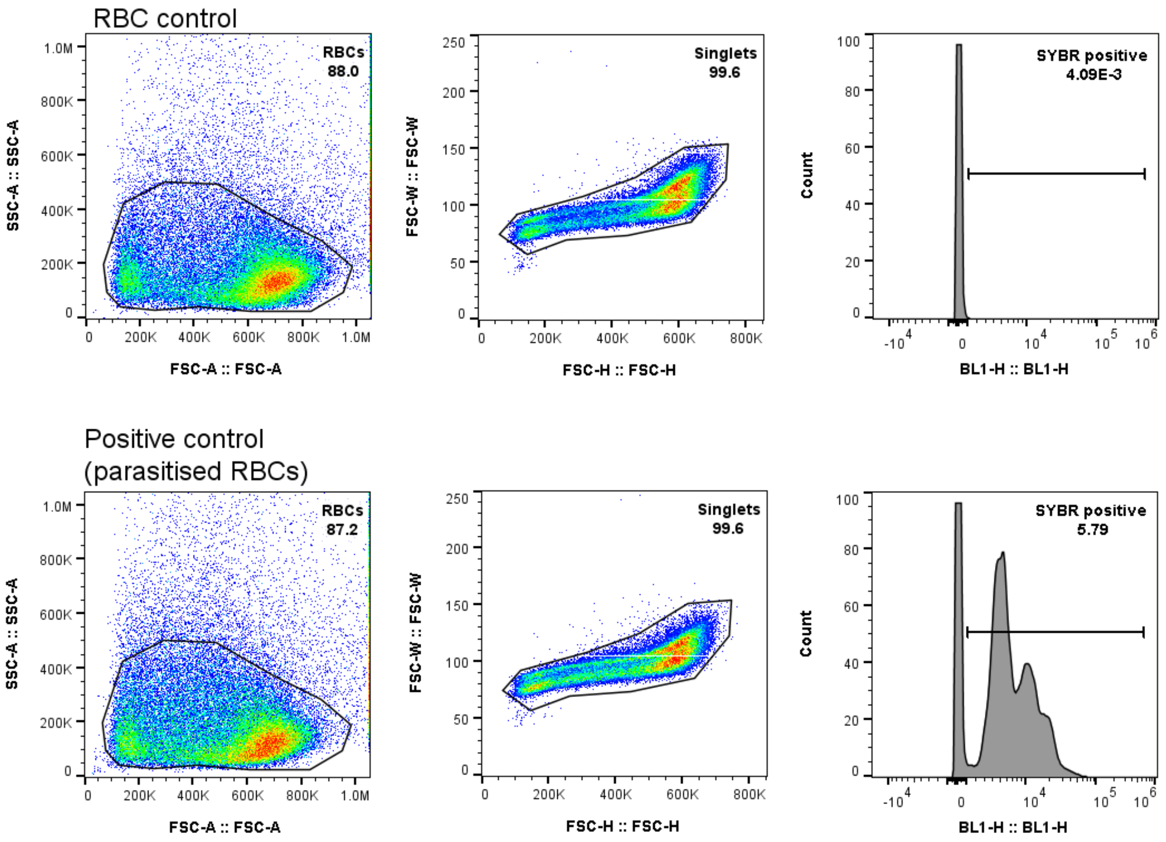

Supplementary Figure 4. Flow cytometry gating strategies used to determine the proportion of SYBR-positive, parasite infected RBCs vs uninfected RBCs. Top panels indicate an RBC only, negative control. Bottom panels depict a sample containing parasitised RBCs. For both, RBCs were gated by plotting side scatter area against forward scatter area (panels in position 1). Doublet discriminated was achieved by gating forward scatter width against forward scatter height (panels in position 2). Gating of SYBR Green positive, infected RBCs was achieved by plotting a histogram against BL1 (488 nm) height using a 530/30 filter (panels in position 3). Parasitaemia was determined by the number of cells identified in gate 3 as a percentage of those in gate 2.

Supplementary Tables

Supplementary Table 1. Primers, DNA templates, and expected PCR product sizes for generating transfection constructs (white cells) and screening transgenic *P. knowlesi* parasites (green cells).

PCR Product	Fwd primer	Rev primer	product size (bp)	Template(s)
mNeonGreen tag				
mNG PCR product	MH297	MH295	836	mNeonGreen plasmid[2]
PkNBPXa-mNG Donor				
HR1	MH227	MH229	514	WT PkA1H1 gDNA
HR2	MH306	MH231	553	WT PkA1H1 gDNA
HR1-mNG fusion	MH227	MH296	1222	HR1 + mNG PCR products
HR1-mNG-HR2 fusion	MH298	MH299	1566	HR1-mNG + HR2 PCR products
'WT' locus	MH835	MH1128	863	gDNA from transfection
Integrated locus	MH835	MH291	973	gDNA from transfection
PkDBPα-mNG Donor				
HR1	MH221	MH223	524	Recodonised PkDBPα gDNA [1]
HR2	MH224	MH225	521	Recodonised PkDBPα gDNA[1]
HR1-mNG fusion	MH221	MH296	1232	HR1 + mNG PCR products
HR1-mNG-HR2 fusion	MH222	MH226	1637	HR1-mNG + HR2 PCR products
WT locus	FM421	MH225	1046	gDNA from transfection
WT locus	FM421	MH1130	564	gDNA from transfection
Integrated locus	FM421	MH291	691	gDNA from transfection
PkRON2-HA Donor				
HR1	MH847	MH849	1092	WT PkA1H1 gDNA
HR2	MH850	MH851	1007	WT PkA1H1 gDNA
HR1-HR2 fusion	MH848	MH852	1874	HR1 + HR2 PCR products
WT locus	MH890	MH909	1209	gDNA from transfection
Integrated locus	MH890	MH563	1338	gDNA from transfection
PkAMA-1-HA Donor				
HR1	MH841	MH843	872	WT PkA1H1 gDNA
HR2	MH844	MH845	880	WT PkA1H1 gDNA
HR1-HR2 fusion	MH842	MH846	1512	HR1 + HR2 PCR products
WT locus	MH892	MH904	984	gDNA from transfection
Integrated locus	MH892	MH563	996	gDNA from transfection
PkNBPXa N-term LoxP Donor				
HR1	MH953	MH967	1037	WT PkA1H1 gDNA
HR2	MH966	MH957	974	WT PkA1H1 gDNA
HR1-HR2 fusion	MH954	MH958	1651	HR1 + HR2 PCR products
WT locus	MH959	MH1143	1261	gDNA from transfection
Integrated locus	MH959	MH1172	1284	gDNA from transfection
PkNBPXa C-termLoxP + HA tag Donor				
HR1	MH835	MH238	858	WT PkA1H1 gDNA
HR1 extended	MH835	MH837	895	HR1 PCR product
HR2	MH838	MH839	852	WT PkA1H1 gDNA

HR1ext-HR2 fusion	MH975	MH1023	1507	HR1e + HR2 PCR products
WT locus	MH1129	MH1128	1004	gDNA from transfection
Integrated locus	MH1129	MH563	997	gDNA from transfection
PvDBP cKO + HA tag Donor				
C-term LoxP	FM0167	FM1893	618	pDonor_PvDBP^{OR} [3]
C-term LoxP extended 1	FM1895	FM1894	574	C-term LoxP
C-term LoxP extended 2	FM1897	FM1896	247	C-term LoxP extended 1
N-term LoxP	FM0141	MH1788	686	pDonor_PvDBP^{OR} [3]
N-term LoxP extended	FM1899	FM1898	652	N-term LoxP
WT locus	FM1973	FM188	2773	gDNA from transfection
Integrated locus	FM1897	FM1041	980	gDNA from transfection
DiCre Cassette Donor				
Cre59	FM268	FM267	536	pBS_DC_hsp86-Bip5[4]
Cre60	FM265	FM266	1220	pBS_DC_hsp86-Bip5[4]
EF1α 5'UTR	FM269	FM270	646	PkconGFPp230p[4]
PbDT 3'UTR	FM271	FM272	864	PkconGFPp230p[5]
PfHRP2 3'UTR	FM273	FM274	587	PkconGFPp230p[5]
PkHSP70 5'UTR	FM275	FM276	1345	PkconGFPp230p[5]
p230p HR1	FM047	FM053	417	WT PkA1H1 gDNA
P230p HR2	FM089	FM006	431	WT PkA1H1 gDNA
WT locus	FM49	FM50	859	gDNA from transfection
Integrated locus	FM90	FM32	891	gDNA from transfection
NBPXa C-term Guide plasmid				
Piece 1	MH400	MH236	442	pL_11HF
Piece 2	MH235	MH295	704	pL_11HF
Full length insert	FM401	FM285	662	Piece 1 + Piece 2 PCR products

Supplementary Table 2. Sequences of primers used in this study.

Primer	Sequence
MH297	GGATCCGGTGGAGGCAGCGG
MH295	CGACAGGTTTCCCGACTGGAAAG
MH227	GAAAATAAATCTATTAGAGGAAGAGGAAGTTAAGC
MH306	ACAGATGTTATGGGAATGGATGAATTGTATAAATAATGAGTAAGGGGGATAGAAGTTTATACAAAAAG
MH298	CAGTTACTAGCAATTTGAATGAGCAGT
MH229	ACCTCCGCTGCCTCCACCGGATCCTATATATTCGTTACTTTCGTCAAAGGTAACTTCA
MH231	TTCGTTTTTCAAAATATGTCTTTTAGGCACC
MH296	TTATTTATACAATTCATCCATTCCCATAACATCTGT
MH299	AGTTTCTCTGATTATCTAATTAATTGATATAAATTCCCATC
MH221	ACAACCGGAAACTGAACCGG
MH224	GGAATGGATGAATTGTATAAATAATGATGCTACTTGGGTAAGTAAGGAGA

MH222	TTTATCCGCGGAGAAGATCATCCTGATGAGCGAAG
MH223	ACCTCCGCTGCCTCCACCGGATCCGCTGTAGTCAAGGGGGGTGAACTG
MH225	CGGGGCTAATTTGTCCGTGTA
MH226	TTTATGAATTCGACAAATTAATGGCACATTTTTCTCTTTTGG
MH847	TTCAGTGTAGCTAGAAACCCGGG
MH848	GCTGATCATTGTTCTGCCTTGTG
MH851	TGCAAAATCCTCTTGTTTTCCCC
MH852	GGTTCAAAATAGGATAAGTCAAAACGGC
MH841	CAACCGAATCGTTTTCGAAGCTAG
MH842	TGATTAAAAGTGCCTTTCTTCCAGTGG
MH845	TATGGATTATATTAAAGTAAGCGTATACGCCATG
MH846	TAGAGTGCTCATATAAGGCACGCAC
MH850	TACCCATACGATGTTCCAGATTACGCTTAGGGTGGTGACTTGTCCATCTC
MH849	AGCGTAATCTGGAACATCGTATGGGTACATCTGTATGCGGGCGTACGA
MH844	TACCCATACGATGTTCCAGATTACGCTTGAGTGGGGAAGCAACGTATTATTCG
MH843	AGCGTAATCTGGAACATCGTATGGGTAGTAGTAAGGCTTCTCCATCAGAACAG
MH953	CTTGTGGTGCTCTTTTAGGTAAAATTACC
MH954	TTATCCGCGGGAAGCTGTTTGAGGTAATCGTGTG
MH957	CAGCCGAATTGCACTCCTTAG
MH958	TTATGCGGCCGCCCTGTTCGTTACCTTCTGTCTGTG
MH966	CTTCGTATAGCATACATTATACGAAGTTATATGCTAAACACCCCTTTATGTTAAACC
MH967	TCGTATAATGTATGCTATACGAAGTTATAGGATGTATAATAATGCCATATAGATCAG CC
MH835	GGCAAGATAGAAGCATATATCGAAAACATTTC
MH838	ATAGCATACATTATACGAAGTTATGTAAGGGGGATAGAAGTTTATACAAAAAGAAA ATC
MH975	TTATCCGCGGGAATCCTATAATGAAGAGGCAAGGAAAAAATTAC
MH238	AGCGTAATCTGGAACATCGTATGGGTATATATATTCGTTACTTTCGTCAAAGGTAA CTTCA
MH837	ATAACTTCGTATAATGTATGCTATACGAAGTTATTCAAGCGTAATCTGGAACATCGT ATG
MH839	CGTATTTCCCCATAAAGATGAATGCG
MH1023	TTATGCGGCCGCGAAAATTATGAAAACGCCATGTTTAAATTTGC
FM0167	CGAGCGGAGAAAGCACATGAACAA
FM1893	TACGAAGTTATTCAAGCGTAATCTGGAACATCGTATGGGTAGCTGTAGTCCAGGG GGGTG
FM1895	CCTGTCCAACGGAAAGCTGGAC
FM1894	GCGGCCGCATAACTTCGTATAATGTATGCTATACGAAGTTATTCAAGCGTAATCTG GAAC
FM1897	CTGCTGCTGATCGCCAGCAGA
FM1896	CTCCTTACTTACCCAAGTAGCAGCGGCCGCATAACTTCGTATAATGTAT
FM0141	GTTTTCCCAGTCACGAC
MH1788	CTTCGTATAATGTATGCTATACGAAGTTATTGTATTTAAAAATAAGTGTATACG
FM1899	CACTATAGGGCGAATTGGCGGAAG
FM1898	TCTTGCCCTTCATACTAGTATAACTTCGTATAATGTATGCTATACGAAGTTATTG
FM268	ATATGGTACCTCAGTTCAGCTTGCACCAGGCA
FM267	ATATCTCGAGATGGCCCCTAAGAAGAAGAGAAAGG

FM265	ATATCTCGAGATGGCCCCTAAGAAGAAGAGAAAGG
FM266	ATATGGTACCTCAGTCCCCATCCTCCAGCAG
FM047	ATATCCGCGGCAGAAGCCGGGTTAGCAGCAC
FM053	ACTAGTTGGAACCCACTTCGGGTGGTC
FM89	ATATGCTGAGCATGAACCTCTGAGCGAAGAGGAGC
FM006	TTTTACCGTTCCATGGGGCGCGCCACATGGCACTCCTTATTATCCTTCGTTAG
FM269	AGGATATGCGGCCGCTAAGTAACCCTTGCATATGCCCCT
FM270	AGGATATACTAGTATAGGTACCATACTCGAGTTTCGAATAAAATTAAATTGAAAAAA AGGTAAGTACGGG
FM271	ATATGGTACCATATGGCAGCTTAATGTTCGTTTTTCTTATTTATATATTT
FM272	ATATGCTCAGCCTACCCTGAAGAAGAAAAGTCCGATG
FM273	ATATGGTACCATTTAATAATAGATTAAAAATATTATAAAAATAAAAACATAAACACAG AA
FM274	ATATACTAGTTAGATTTAATAAATATGTTCTTATATATAATGAGAAATAAATATTTAA CA
FM275	GGATATGCGGCCGCATGCAATATACCCATTTTGAATACACCCCA
FM276	GGATATGCTCAGCATAGGTACCATACTCGAGTTTTACGGGGATCTGCAAGGGG
MH400	TATGCCTAAGATCGTCTCCCCTTTGATT
MH236	TTCTAGCTCTAAAACTACTCATATATATTCGTTACAATAATATACTGTAA
MH235	TTACAGTATATTATTGTAACGAATATATATGAGTAGTTTTAGAGCTAGAA
MH295	CGACAGGTTTCCCGACTGGAAAG
FM401	CATTGTTCCCCCCTTTGTTTTGCAAG
FM285	TACTTATGCGTATACAAAGCCTTCTTCAC
MH835	GGCAAGATAGAAGCATATATCGAAAACATTTC
MH291	TTGGATTTCCTGTTCCTTGTCCAACC
FM421	TTCATGCGGAACAGCAACAG
MH225	CGGGGCTAATTTGTCCGTGTA
MH1130	ACCCAAGTAGCATCAGCTGTAG
MH890	TGC TAG AAA AAG TGG CAG AGT TAC ATA AG
MH909	AGT CAC CAC CCT ACA TCT GTA TGC
MH563	CGTAATCTGGAACATCGTATGGG
MH892	GTA ATG ATT GGG AAA ACA AGT GCC C
MH904	GCT TCC CCA CTC AGT AGT AAG G
MH959	CCCCTTTCCCAGGAACAAATTG
MH1143	CATAAAGGGGTGTTTAGCATAGGATG
MH1172	GTTTAGCATATAACTTCGTATAATGTATGCTATACGAAG
MH1129	GAAGGCATATACGAAATATGGAAAAGAGC
MH1128	CTTTTTGTATAAACTTCTATCCCCCTTACTCATATATATTC
FM1973	CCATGTACACGATTTGTGTACTTATAGAATC
FM188	AGGAGCACCTGATTGAGAACCTGGA
FM1897	CTGCTGCTGATCGCCAGCAGA
FM1041	GTAGGGAACATTTCTTTCTGCGG
FM49	GAAGATTCCGCAAAGCTTTGTCGGTTA
FM50	ACGCTATGGAAGCAGTTGTCTGGAT
FM90	TGGGAAATACAGGAAATAACGGTGTTATGT
FM32	CCTAATCATGTAAATCTTAAATTTTTCTTTTTAAACATATG

Supplementary References:

1 Mohring F, Hart MN, Rawlinson TA, Henrici R, Charleston JA, Diez Benavente E *et al.* Rapid and iterative genome editing in the malaria parasite Plasmodium knowlesi provides new tools for P. vivax research. *Elife* 2019; **8**: 1–29.

2 Yahata K, Hart MN, Davies H, Asada M, Wassmer SC, Templeton TJ *et al.* Gliding motility of Plasmodium merozoites. *Proc Natl Acad Sci U S A* 2021; **118**: e2114442118.

3 Rawlinson TA, Barber NM, Mohring F, Cho JS, Kosaisavee V, Gérard SF *et al.* Structural basis for inhibition of Plasmodium vivax invasion by a broadly neutralizing vaccine-induced human antibody. *Nat Microbiol* 2019; **4**: 1497–1507.

4 Collins CR, Das S, Wong EH, Andenmatten N, Stallmach R, Hackett F *et al.* Robust inducible Cre recombinase activity in the human malaria parasite Plasmodium falciparum enables efficient gene deletion within a single asexual erythrocytic growth cycle. *Mol Microbiol* 2013; **88**: 687–701.

5 Moon RW, Hall J, Rangkuti F, Shwen Y, Almond N, Mitchell GH. Adaptation of the genetically tractable malaria pathogen Plasmodium knowlesi to continuous culture in human erythrocytes. *PNAS* 2013; **110**: 531–536.
