# Supplementary material for: Sequential roles for red blood cell binding proteins enable phased commitment to invasion for malaria parasites
Source: Nat Commun. 2023 Aug 1;14:4619. doi: 10.1038/s41467-023-40357-z (PMC10393984; doi:10.1038/s41467-023-40357-z)
Supplement: Supplementary file 13 — Reporting Summary [file 41467_2023_40357_MOESM13_ESM.pdf]

nature portfolio

Corresponding author(s): *Double-blind peer review submissions: write DBPR and your manuscript number here instead of author names.*

Last updated by author(s): *YYYY-MM-DD*

Reporting Summary

Nature Portfolio wishes to improve the reproducibility of the work that we publish. This form provides structure for consistency and transparency in reporting. For further information on Nature Portfolio policies, see our Editorial Policies and the Editorial Policy Checklist.

Statistics

For all statistical analyses, confirm that the following items are present in the figure legend, table legend, main text, or Methods section.

n/a	Confirmed	
☐	☒	The exact sample size (*n*) for each experimental group/condition, given as a discrete number and unit of measurement
☐	☒	A statement on whether measurements were taken from distinct samples or whether the same sample was measured repeatedly
☐	☒	The statistical test(s) used AND whether they are one- or two-sided<br>*Only common tests should be described solely by name; describe more complex techniques in the Methods section.*
☒	☐	A description of all covariates tested
☐	☒	A description of any assumptions or corrections, such as tests of normality and adjustment for multiple comparisons
☐	☒	A full description of the statistical parameters including central tendency (e.g. means) or other basic estimates (e.g. regression coefficient) AND variation (e.g. standard deviation) or associated estimates of uncertainty (e.g. confidence intervals)
☐	☒	For null hypothesis testing, the test statistic (e.g. *F*, *t*, *r*) with confidence intervals, effect sizes, degrees of freedom and *P* value noted<br>*Give P values as exact values whenever suitable.*
☒	☐	For Bayesian analysis, information on the choice of priors and Markov chain Monte Carlo settings
☒	☐	For hierarchical and complex designs, identification of the appropriate level for tests and full reporting of outcomes
☐	☒	Estimates of effect sizes (e.g. Cohen's *d*, Pearson's *r*), indicating how they were calculated

Our web collection on statistics for biologists contains articles on many of the points above.

Software and code

Policy information about availability of computer code

Data collection	Parasite growth assays were analysed by flow cytomentry on an Attune NxT flow cytometer. Live cell microscopy and immunofluorecence assays were performed using an inverted microscope (Ti-E; Nikon) with NIS-Element Advanced Research imaging software (Nikon, V5.30)
Data analysis	Microscopy images were processed and statistically analysed using the NIS Advanced Research software package (V5.30). Flow cytometry data were analysed using FACSDiva 6.1.3 software. Data resulting from live cell imaging experiments was statistically analysed using Prism (version 10).

For manuscripts utilizing custom algorithms or software that are central to the research but not yet described in published literature, software must be made available to editors and reviewers. We strongly encourage code deposition in a community repository (e.g. GitHub). See the Nature Portfolio guidelines for submitting code & software for further information.

Data

Policy information about availability of data

All manuscripts must include a data availability statement. This statement should provide the following information, where applicable:

- Accession codes, unique identifiers, or web links for publicly available datasets
- A description of any restrictions on data availability
- For clinical datasets or third party data, please ensure that the statement adheres to our policy

P. knowlesi normocyte binding protein Xa (NBPXa), Duffy binding protein alpha (PkDBPα), Apical membrane antigen 1 (AMA-1), and Rhoptry neck protein 2 (RON2) constructs were derived from P. knowlesi strain H sequences from the PlasmoDB Database (www.plasmodb.org) with respective accession numbers:

PKNH_1472300, PKNH_0623500, PKNH_0931500, and PKNH_1230100. All other data generated in this study are provided in the Supplementary Information/ Source Data file

Human research participants

Policy information about studies involving human research participants and Sex and Gender in Research.

Reporting on sex and gender	N/A
Population characteristics	N/A
Recruitment	N/A
Ethics oversight	N/A

Note that full information on the approval of the study protocol must also be provided in the manuscript.

Field-specific reporting

Please select the one below that is the best fit for your research. If you are not sure, read the appropriate sections before making your selection.

- [x] Life sciences
- [ ] Behavioural & social sciences
- [ ] Ecological, evolutionary & environmental sciences

For a reference copy of the document with all sections, see nature.com/documents/nr-reporting-summary-flat.pdf

Life sciences study design

All studies must disclose on these points even when the disclosure is negative.

Sample size	Sample sizes for each experiment were chosen to be consistent with the field norms. E.g., for flow cytometry experiments, at least three biological replicates, each with three technical replicates for each data point. FACS analyses to determine parasitaemia counts routinely measured the numbers of parasite-infected cells in a total of 50,000 red blood cells. Since parasitaemia values ranged from 0.1-15%, this was considered sufficient to provide statistically robust measurements of parasitaemia. For imaging, typically interactions resulting from 10-20 schizont egresses were analysed– e.g., as per 10.1371/journal.ppat.1004670. Specific values (e.g., number of merozoite-RBC contacts analysed per condition) are provided in the figures, figure legends and/or methods under the 'Statistics and Reproducibility' section.
Data exclusions	No data were excluded from this study
Replication	For all live cell imaging experiments, at least 2-3 independent repeats were performed per condition. For quantification of parasite replication by flow cytometry, 3-5 independent experiments, each with 3 technical repeats, were performed. For quantification of parasite growth by LDH assay, 2-3 independent experiments were performed. Westerns, PCRs, and IFAs demonstrating PkNBPXa and PvDBPa cKO status were repeated independently 3 times. For PkNBPXa and PkDBPα secretion assays, 2 biological repeats were performed. For all experiments, all attempts at replication were successful.
Randomization	Randomization was not relevant to this study as no subjective judgements were required about which data to include, exclude, or measure.
Blinding	The investigators were not blinded during the experiment and/or when assessing the outcome. For growth assays, analysis were performed on quantitative endpoints that are subject to minimal investigator bias. Several live cell imaging experiments (eg. determining how many merozoites/schizont invaded BAPTA-treated red blood cells), also had clearly defined end points. For all other live cell imaging experiments, every effort was made to reduce investigator bias by identifying and clearly defining measurable, quantitative parameters (eg defining a deformation strength scoring system or quantifying the length of time a merozoite spent deforming a RBC) prior to the start of analysis and ensuring all relevant controls were in place.

Reporting for specific materials, systems and methods

We require information from authors about some types of materials, experimental systems and methods used in many studies. Here, indicate whether each material, system or method listed is relevant to your study. If you are not sure if a list item applies to your research, read the appropriate section before selecting a response.

Materials & experimental systems

n/a	Involved in the study
☐	☒ Antibodies
☐	☒ Eukaryotic cell lines
☒	☐ Palaeontology and archaeology
☒	☐ Animals and other organisms
☒	☐ Clinical data
☒	☐ Dual use research of concern

Methods

n/a	Involved in the study
☒	☐ ChIP-seq
☐	☒ Flow cytometry
☒	☐ MRI-based neuroimaging

Antibodies

Antibodies used

Primary:
1) Rabbit anti-Normocyte binding protein Xa (generated from recombinant NBPXa - see below)
2) Rat anti-HA (3F10 clone, product code 11867423001, Sigma)
3) Human anti-DARC (2C3clone, product code Ab00893-10.0, Absolute Antibody)
4) Mouse anti-mNeonGreen (32F6 clone, product code 32f6, ChromoTek)
5) Rabbit anti-PkMSP1 (polyclonal, generated from recombinant PkMSP1_19 and described in https://doi.org/10.1073/pnas.1522469113)
6) Rat anti-PfHSP70 (polyclonal, generated against recombinant P. falciparum HSP70 and described in 10.1126/sciadv.abe5396)
7) Mouse monoclonal anti-His (His-Tag mAb, 70796, EMD-Milipore).

Secondary:
8) Donkey anti-Mouse IgG (H+L) Highly Cross-Adsorbed Secondary Antibody, Alexa Fluor™ 488 (Invitrogen)
9) Goat anti-Rabbit IgG (H+L) Highly Cross-Adsorbed Secondary Antibody, Alexa Fluor™ 488 (Invitrogen)
10) IRDye® 680RD Goat anti-Rat IgG Secondary Antibody (LICOR)
11) Goat anti Rat IgG (H/L):HRP (Biorad)
12) Goat anti-rabbit IgG (H/L):HRP (Biorad)

Validation

1) Rabbit anti-PkNBPXa antibodies were raised (Covalab) against recombinant NBPXa (amino acids 151 to 467) and subsequently purified via immunoaffinity to recombinant protein. Antibody subsequently validated by western blot against parasite lysates. Little to no background signal is observed when probed against lysates from NBPXa null parasites.

2) Rat anti-HA is a commercially available antibody from: https://www.sigmaaldrich.com/GB/en/product/roche/roahaha?gclid=EAIaIQobChMIwaulgJPO-gIVA853Ch1MLAOkEAAYASAAEgIOMvD_BwE&gclsrc=aw.ds
"Anti-HA High Affinity is a monoclonal antibody to the HA-peptide (clone 3F10). Anti-HA High Affinity recognizes the HA peptide sequence (YPYDVPDYA), derived from the influenza hemagglutinin protein. The antibody recognizes its antigenic determinant even when the HA peptide epitope is introduced into unrelated recombinant proteins by a technique known as "epitope tagging"." This antibody has been validated for detecting tagged proteins by western and IFA in P. knowlesi in the following publication: https://doi.org/10.7554/eLife.45829

3) Human anti-DARC is a commercially available antibody that binds specifically to the pentapeptide FEDVW of the Duffy receptor. Available from: https://absoluteantibody.com/product/anti-darc-2c3/Ab00893-10.0_human_igg1/bulk. This antibody has been validated by western blot and IFA. The Fy6 epitope it binds to has been demonstrated to block PvDBP and PkDBPa from binding to DARC on human RBCs: 10.1007/s00018-010-0387-6, 10.21769/BioProtoc.3522; 10.1084/jem.166.3.776.

4) Mouse anti-mNeonGreen is a commercially available antibody from: https://www.ptglab.com/products/mNeonGreen-antibody-32F6.htm. Specificity against recombinant mNeonGreen and proteins tagged with mNeonGreen has been determined by western blot and IFA.

5) Generation of rabbit anti-PkMSP1 (raised against recombinant P. knowlesi MSP1) and validation by IFA (probing P. knowlesi schizonts) is described in the following manuscript: https://doi.org/10.1073/pnas.1522469113.

6) Rat anti-PfHSP70 has been validated by western blot, using P. falciparum parasite lysates (10.1126/sciadv.abe5396). This antibody also recognizes P. knowlesi HSP70 (expected size ~74 kDa) by western blot using P. knowlesi parasite lysates (demonstrated in this manuscript).

7) Mouse anti-His is a commercially available antibody from: https://www.merckmillipore.com/GB/en/product/HisTag-Monoclonal-Antibody,EMD_BIO-70796. This antibody has been validated by western blot and IFA for specificity against His-tag. Negligible cross-reactivity with bacterial, yeast, insect, or mammalian cell lysates.

Eukaryotic cell lines

Policy information about cell lines and Sex and Gender in Research

Cell line source(s)

Pk A1-H.1 (https://doi.org/10.1073/pnas.1216457110); HEK293E (Durocher et al., 2002) or HEK293-6E (Loignon et al., 2008) were kindly provided by Yves Durocher (NRC, Montreal)

Authentication	Pk A1-H.1 has been authenticated by whole genome sequencing; Neither the HEK293E nor HEK293-6E cell lines were authenticated
Mycoplasma contamination	All lines tested negative for mycoplasma contamination
Commonly misidentified lines (See ICLAC register)	No commonly misidentified cell lines were used in this study.

Flow Cytometry

Plots

Confirm that:

- [x] The axis labels state the marker and fluorochrome used (e.g. CD4-FITC).
- [x] The axis scales are clearly visible. Include numbers along axes only for bottom left plot of group (a 'group' is an analysis of identical markers).
- [x] All plots are contour plots with outliers or pseudocolor plots.
- [x] A numerical value for number of cells or percentage (with statistics) is provided.

Methodology

Sample preparation	To determine the multiplication rate of mutant parasites, ring stage DMSO and rapamycin-treated cultures were adjusted to a 0.5% parasitaemia and 2% haematocrit and were grown in triplicate in 96 well plates in a gassed chamber at 37 degrees C. After 24 hours, a starting sample was taken and stained with SYBR Green I (1/5000 in PBS; Life Technologies) before measuring parasitaemia by flow cytometry (FACS). A second sample was taken 26 hours later, and a final sample was taken roughly 26 hours later again.
Instrument	Data were collected on an Attune NxT flow cytometer using FACSDiva 6.1.3 software.
Software	Data were collected using FACSDiva 6.1.3 software, and were analysed using FlowJo version 10. Graphpad Prism v10 was used for statistical analysis.
Cell population abundance	At least 50,000 singlets were counted/sample.
Gating strategy	RBCs were gated by plotting side scatter area against forward scatter area. Doublet discriminated was achieved by gating forward scatter width against forward scatter height. Gating of SYBR Green positive, infected RBCs was achieved by plotting a histogram against BL1 (488 nm) height using a 530/30 filter. Parasitaemia was determined by the number of cells identified in gate 3 as a percentage of those in gate 2.

- [x] Tick this box to confirm that a figure exemplifying the gating strategy is provided in the Supplementary Information.
